# Relationships Between Religiosity and Naturally Occurring Social Interaction

**DOI:** 10.1007/s10943-020-01079-4

**Published:** 2020-09-02

**Authors:** John B. Nezlek

**Affiliations:** 1 Institute of Psychology, SWPS University of Social Sciences and Humanities, Kutrzeby 10, 61-719 Poznan, Poland; 2 Department of Psychological Sciences, College of William & Mary, Box 8795, Williamsburg, VA 23185 USA

**Keywords:** Social interaction, Daily diary, Religiosity, Well-being

## Abstract

For 2 weeks, participants (282 US collegians) used a diary technique to describe the social interactions they had each day. These descriptions included how enjoyable the interaction was, how confident they felt, and how intimate the interaction was. They also completed a measure of Allport’s Intrinsic–Extrinsic religious orientation, the Christian Orthodoxy scale, and the Beck Depression Inventory. A series of multilevel modeling analyses found that enjoyment and confidence in social interaction were positively related to the Extrinsic Personal factor of the IE scale, whereas intimacy of interactions was not related to any of these measures. These relationships remained after controlling for orthodox beliefs and depression. These results suggest that the extent to which people find comfort in religious beliefs and practices (e.g., prayer) is positively related to the quality of their daily social experiences.

## Introduction

A considerable body of research indicates that well-being, defined in various ways, is positively related to the quality of one’s social life (Cacioppo and Patrick 2008; Perlman and Vangelisti 2006). A parallel body of research indicates that religiosity is also positively related to well-being (Delle Fave et al. 2013; Newman and Graham 2018). Taken together, these two bodies of research suggest that the quality of one’s social life should be positively related to religiosity.

By and large, the existing research supports such a conclusion (e.g., Lim and Putnam 2010), and the present study was designed to complement existing research. Participants in the present study provided measures of religiosity and of the quality of their social lives, and the general expectation was that more religious people would have more positive social lives than less religious people. We consider these relationships in more detail in the discussion section.

In their review of research on religiosity and well-being, Newman and Graham (2018) noted that: “In many of these studies, religiosity has typically been measured with a single item, such as “Is religion an important part of your daily life?” or “Thinking about your life these days, how often do you attend religious services, apart from social obligations such as weddings or funerals?” Although such measures could be informative in predicting well-being, specific religious beliefs and practices could relate to well-being in nuanced ways.”

Consistent with their implicit suggestion that religiosity should be measured with something more nuanced than single items, the present study operationalized religiosity in terms of multiple constructs each of which was measured with multiple items. Religiosity was defined in terms of Allport’s classic distinction between Intrinsic and Extrinsic Orientations, and given the nature of the sample studied, religiosity was also defined in terms of the acceptance of orthodox Christian beliefs.

Although Newman and Graham (2018) did not discuss this, the existing research on relationships between religiosity and well-being also suffers from important shortcomings in terms of how well-being has been conceptualized. One shortcoming is that the roles of sociality have not been studied explicitly. Social life is an important foundation for well-being, and without measuring people’s satisfaction with their social lives per se, it cannot be known how important the quality of people’s social lives is in terms of understanding relationships between religiosity and well-being.

In addition, in studies of relationships between religiosity and well-being, well-being has been measured primarily using single occasion assessments, i.e., asking people how they feel about their lives only once. Although such assessments can be valuable, as discussed by Nezlek (2012, pp. 4–6) they can be influenced by various factors that might undermine their validity such as the greater salience of more recent events compared to the salience of more temporally distant events or the “lumping together” of distinct influences due to difficulties in recalling specific occasions/events. In the present study, such influences were minimized because participants described the individual social events that constituted their lives and did so soon after they had occurred.

The lack of specificity in existing research and theory regarding relationships between religiosity and social experience (broadly defined) did not provide a basis for hypotheses involving specific measures of either construct. Nevertheless, the available research did suggest that, broadly speaking, religiosity should be positively related to the quality of people’s daily social interactions. Moreover, given previous research that found a positive relationship between the social support people experienced in social interaction and the positive emotions they had (e.g., Kafetsios and Nezlek 2012), I expected the extent to which people viewed their faith as a source of support would be positively related to the quality of their social interactions.

## Methods

### Participants

Participants were 282 undergraduates attending an American university (164 women, *M*_age_ = 19.7, SD = 1.19). They volunteered to participate in a study about daily social interaction in response to an announcement made in their classes. Participants were paid $20 upon completion of the study.

### Measures

Social interaction was measured using a variant of the Rochester Interaction Record (RIR; Wheeler and Nezlek 1977). Similar to previous studies using the RIR, participants used a fixed format record to describe the social interactions they had. For each interaction, they indicated whom they were with by using unique initials for each person who was present, and they indicated the sex of the other people. If more than 4 others were present, no individual initials were listed and the interaction was described as a group interaction. The length of each interaction was also reported. Participants also rated each interaction in terms of how enjoyable the interaction was, how confident they felt, and how intimate the interaction was. These ratings were made using 9-point scales, labeled 1 = not, 3 = slightly, 5 = somewhat, 7 = quite, and 9 = very.

These ratings of interaction were meant to measure what have become to be known as the “Big Two” of interpersonal judgment and behavior: agency and communion (e.g., Abele and Wojciszke 2013). Confidence/competence was intended to measure agency, which is usually defined in terms of goal achievement and task functioning. Intimacy was intended to measure communion, which is usually defined in terms of the maintenance of relationships and social functioning. Enjoyment was included because the hedonic dimension (bad–good) has long been recognized as a central dimension of human experience (Osgood et al. 1957).

Religiosity was measured using the Intrinsic–Extrinsic Orientation Scale Revised (IE-R; Gorsuch and McPherson 1989). The IE-R has three subscales: *Intrinsic Orientation* (e.g., My whole approach to life is based on my religion), *Extrinsic Personal Orientation* (e.g., What religion offers me most is comfort in times of trouble and sorrow), and *Extrinsic Social Orientation* (e.g., I go to church mostly to spend time with my friends). Participants also completed the short version (six items) of the Christian Orthodoxy scale (CO; Hunsberger 1989), for example “Jesus Christ is the divine Son of God.” Participants responded to the items on the IE-R and CO scales using 9-point scales (1 = strongly disagree, 9 = strongly agree). Finally, participants completed the 21-item Beck Depression Inventory (BDI; Beck 1967) using the standard 4-point scale (0 through 3).

### Procedure

During an introductory meeting, the importance of understanding social interaction was explained, and participants’ roles as co-investigators were emphasized. Participants were told that the study concerned people’s patterns of daily social life and that they would use a structured form to describe their social interactions. The instructions were a variant of those described by Nezlek (2012, pp. 133–138). At this meeting, participants also completed the IE-R, the CO, and BDI scales.

Participants were told to use the RIR to record every social interaction they had that lasted 10 min or longer during a 2-week period. Consistent with previous research, interactions were defined as encounters with other people in which the participants attended to one another and adjusted their behavior in response to one another. Examples were provided to clarify what was an interaction, e.g., having lunch with someone, and what was not, such as sitting next to a stranger while studying and not talking to that person.

The response categories on the RIR were discussed until participants understood their definitions and knew how to use the forms. Enjoyment was defined as “how pleasurable or satisfying” participants found each interaction to be. Intimacy was defined as “how interpersonally close” an individual felt to his or her cointeractants, with specific mention that “intimacy does not have to be sexual, nor does it have to be evident only through conversation.” Confidence was defined as “how self-assured you were and how competent you felt.”

Participants were told to complete the records at least once a day at a uniform time, such as before going to bed. They were told that if they missed a day, they should not try to recall it but should add days to the end of the study. Participants were given an instruction booklet and enough interaction forms for the duration of the study. After 3 days, participants were contacted to see whether they were having any problems; none was reported.

After they finished maintaining, the diary participants were interviewed individually about the difficulties and potential sources of inaccuracy in their data. They were encouraged to be candid when describing how they maintained the diary, and they were told they would be paid regardless of what they said about how they maintained their diaries. Participants reported that their diaries were accurate representations of their social lives (*M* = 7.29, 1–9 scale) and that maintaining the diary was not difficult (*M* = 3.41, 1–9 scale). Consistent with instructions, they reported updating their diaries an average of 1.79 times per day, and on average, they reported spending 14.6 min doing this. Participants maintained the diary for on average of 14.5 days (4082 total days), and on average, participants recorded 83.0 interactions over the course of the study (23397 total interactions). All data described in this paper are available at https://osf.io/pyusm/?view_only=052cd89434dd419c81fb4e3c22ae74e0.

## Results

### Psychometric Properties of Individual Difference Measures

The measures of religiosity and depression were scored following established guidelines, and summary statistics for these measures are presented in Table 1. A few statistics from this table are noteworthy. First, all scales were sufficiently reliable so that they could be treated as measures of their respective constructs. Moreover, the analyses did not indicate that the reliability of any scale could be improved by deleting an item. Second, different numbers of respondents answered different items that constitute the subscales of the IE-R. These differences reflect the fact that participants were told that they did not have to answer questions that were not relevant to their experience. Third, correlations between the measures of religiosity were similar to those reported in previous research using similar samples (e.g., Kirkpatrick 1993). Fourth, BDI scores were not significantly related to any measure of religiosity.

**Table 1:** Descriptive statistics for and correlations between trait-level measures

	*M*	SD	*α*	*N*	EPer	ESoc	Orth	BDI
Intrinsic	5.06	1.90	.86	282	.36**	.16*	.57**	− .08
Extrinsic Personal	5.36	1.89	.77	270		.20**	.52**	.10
Extrinsic Social	3.12	1.94	.86	246			.16*	.00
Orthodoxy	6.86	2.29	.94	280				− .02
BDI	6.44	6.11	.88	282				

*EPer* Extrinsic Personal Orientation; *ESoc* Extrinsic Social Orientation, *Orth* Christian Orthodoxy, *BDI* Beck Depression Inventory

*p < .05; **p < .01

### Relationships Between Religiosity and Reactions to Social Interaction

Relationships between religiosity and measures of social interaction were examined with a series of multilevel models (MLM) in which interactions were treated as nested within persons. See Nezlek (2003, 2012) for descriptions of using MLM to analyze social interaction diary data. The basic model is provided below. Such a model is called an “unconditional model” because there are no predictors at any level of analysis, and unconditional models provide the basic summary statistics: an estimate of the mean and the variance at each level of analysis.

In the analyses, *y* is a rating of an interaction, and *i* interactions are nested within *j* persons. In the model below, a mean (*β*_0*j*_) is estimated for each of *j* persons, and the overall mean is *γ*_00_. The within-person variance (how much do measures of interactions vary) is the variance of *r*_*ij*_, and the between-person variance (how much do the means of people vary) is the variance of *μ*_0*j*_:

$${\text{Within-person:}}\quad y_{ij} = \beta_{0j} + \, r_{ij} .$$

$${\text{Between-person:}}\quad \beta_{0j} = \gamma_{00} + \mu_{0j}$$

Summary statistics for the three ratings of interactions are presented in Table 2. As can be seen from these statistics, on average, participants found their interactions to be enjoyable and they tended to feel confident when they were with other people. Average intimacy of interactions was toward the midpoint of the scale, a finding consistent with previous research (e.g., Tidwell et al. 1996). Moreover, the variance estimates indicated that there was sufficient variance at the between-person level to justify examining person-level relationships between religiosity and ratings of interactions.

**Table 2:** Descriptive statistics of ratings of interactions

	Mean	Variance	
		Within	Between
Enjoyment	6.71	2.46	.52
Confidence	7.01	1.76	.88
Intimacy	4.89	3.65	2.53

Relationships between religiosity and ratings of interactions and between depression and ratings of interactions were examined by adding predictors to the between-person model presented above. Initially, each of the measures of the religiosity and BDI scores was analyzed separately. The model is presented below. The statistical significance of relationships between a rating of interaction and a person-level measure was estimated via the significance of the *γ*_01_ coefficient. MLM analyses estimate only unstandardized coefficients, and to ease the interpretation of the results, person-level measures were standardized prior to analysis. This meant that the *γ*_01_ represented the change in a person’s mean rating of interaction associated with a 1 SD increase in a person-level measure:

$${\text{Between-person:}}\quad \beta_{0j}= \gamma_{00} + \gamma_{01} * ( {\text{Person-level measure}} ) + \mu_{0j}$$

The results of these analyses are summarized in Table 3. As can be seen from these coefficients, all of the person-level measures were significantly related to how enjoyable interactions were (positively for measures of religiosity and negatively for *BDI* scores), none of the person-level measures were significantly related to intimacy of interactions, and only *Extrinsic Personal Orientation* and *BDI* scores were significantly related to confidence in interaction (positively and negatively, respectively).

**Table 3:** Relationships between trait measures and reactions to interactions

	Intrinsic	E-Personal	E-Social	Orthodoxy	BDI
Zero-order relationships, each predictor considered separately					
Enjoyment	.12**	.14**	.10*	.09*	− .16**
Confidence	.06	.16**	.02	.10^a^	− .19**
Intimacy	.09	.14	.12	.12	.08
Coefficients when all predictors analyzed together					
Enjoyment	.09^a^	.16**	.07	− .06	− .16**
Confidence	− .06	.24**	− .02	− .01	− .21**
Intimacy	.04	.03	.09	.10	.13

*p < .05; **p < .01, ^a^*p* < .10

As indicated by the correlations presented in Table 1, measures of religiosity were positively correlated. This meant that the relationships represented by the coefficients presented in Table 3 may not have represented unique relationships (unshared variance) between enjoyment and measures of religiosity. This possibility was examined by including all measures of religiosity as predictors in one analysis.

The results of these analyses were quite clear. When all measures of religiosity were analyzed together, only the relationships between enjoyment and *Extrinsic Personal Orientation* remained significant at the .05 level (*γ*_02_ = .16, *p* < .01), although the relationship between enjoyment and *Intrinsic Orientation* approached conventional levels of significance (*γ*_01_ = .09, *p* = .09). In the analyses of confidence in interaction, the only coefficient that was significant (or near significant) was *Extrinsic Personal Orientation* (*γ*_01_ = .24, *p* < .01). Given that *BDI* scores were uncorrelated with religiosity, including BDI scores in these analyses did not change the statistical significance of the coefficients for *Extrinsic Personal Orientation* in any analysis.

When Wheeler and Nezlek (1977) introduced the RIR, they highlighted the importance of taking sex differences into account when examining social interaction. They suggested that this could be done in two ways: in terms of the “sexual composition” of an interaction (where the others present the same sex as the participant, the opposite sex, or where members of both sexes are present) and in terms of the sex of the person keeping the diary (men vs. women). Taking sexual composition into account requires adding predictors representing each type of interaction to the within-person model, and taking the sex of the participant into account requires adding sex as a predictor to the person-level model. See Nezlek (2003) for a discussion of such analyses. Additional analyses that considered these possibilities did not find that relationships between religiosity and ratings of interactions varied as a function of the sexual composition of interactions or as a function of the sex of the participant.

### Relationships Between Religiosity and Quantity of Social Interaction

The preceding analyses focused on people’s reactions to interactions, but as discussed by Nezlek (2001b), reactions to interactions (quality) are distinct from how socially active people are (quantity of interaction). Accordingly, a series of analyses were done that examined relationships between religiosity and quantity of social activity. In these analyses, days (*n* = 4082) were treated as nested within persons (*n* = 282), and social activity was defined in terms of number of interactions per day, measured for all interactions and separately for same-, opposite-, and mixed-sex interactions. As in the previous analyses, measures of religiosity and *BDI* scores were standardized prior to analysis.

These analyses found only three significant or marginally significant relationships between individual differences in social activity and religiosity. First, the number of same-sex interactions per day was positively related to the strength of Christian orthodox beliefs (*γ*_01_ = .18, *p* < .05). Second, the total number of interactions per day was negatively related (marginally significant) to scores on the *External Social Orientation* scale (*γ*_01_ = − .23, *p* < .08). Third, the number of opposite-sex interactions per day was negatively related to scores on the *External Social Orientation* scale (*γ*_01_ = − .12, *p* = .05).

Total number of interactions per day included opposite-sex interactions per day. Given these results, a follow-up analysis was conducted to determine whether the relationship between *External Social Orientation* and total number of interactions per day reflected the relationship between *External Social Orientation* and number of opposite-sex interactions per day. This was done by analyzing the relationship between *External Social Orientation* and total number of interactions per day controlling for individual differences in the number of opposite-sex interactions per day.

In this analysis, the relationship between *External Social Orientation* and total number of interactions per day was not significant day (*γ*_11_ = − .03, *p* = .38). This indicates that the zero-order relationship between *External Social Orientation* and total number of interactions per day was due to relationships between *External Social Orientation* and number of opposite-sex interactions per day. Such a conclusion is also consistent with the lack of a significant relationship between *External Social Orientation* and the number of same-sex interactions per day (*γ*_01_ = − .03, *p* = .75) and mixed-sex interactions per day (*γ*_01_ = − .07, *p* = .12).

## Discussion

The analyses found that religiosity, defined in terms of Allport’s classic typology of Intrinsic and Extrinsic Orientations, was positively related to three important aspects of daily social life: how enjoyable people found their time with others, how confident and self–assured they felt when they were with other people, and how much contact they had with the opposite sex. *Intrinsic Orientation* and *External Social Orientation* were positively related to how enjoyable people found their social interactions to be, and *Personal Extrinsic Orientation* was positively related to enjoyment of interactions and to how confident and self-assured people felt. *External Social Orientation* was also negatively related to amount of opposite-sex contact. In addition, the extent to which people held orthodox Christian beliefs was positively related to how enjoyable their interactions were and to the amount of same-sex contact they had each day.

When evaluating these results, it is important to keep in mind that the social milieu in which the study was conducted was primarily Christian. Although the university at which the study was conducted is not affiliated with a specific religious group (nor has it ever been), the majority of participants described themselves as Christian of some type (228 of 282). Moreover, the full sample average score on the *Christian Orthodoxy* scale was 6.87 (maximum score 9).

There is an established body of research indicating that similarity leads to attraction (e.g., McPherson et al. 2001), and the dominant explanation for this relationship is that attitudinal similarity is rewarding (e.g., Byrne and Nelson 1965). Individuals with higher scores on the *CO* scale were probably more likely to interact with attitudinally similar others than individuals with lower scores on the *CO* scale, which would lead to more satisfying interactions.

A connection between religiosity and social life is also suggested by the positive relationship between enjoyment and *External Social Orientation* (e.g., “I go to church mainly because I enjoy seeing people I know there”). These items clearly focus on the social rewards associated with being an active practitioner. If religious similarity serves as a basis for friendship formation, then individuals for whom attending religious services serves an important social function should find interacting with their fellow practitioners (or congregants) more rewarding than individuals for whom attending services does not serve a social function so strongly. Unfortunately, the religious affiliation of the people with whom participants interacted was not measured, so the preceding explanations are speculative.

Regardless, when relationships between enjoyment and *Intrinsic Orientation, External Social Orientation,* and Christian Orthodoxy were controlled for *Extrinsic Personal Orientation*, these relationships became nonsignificant. Moreover, *Extrinsic Personal Orientation* was also positively related to confidence in interaction, whereas the other measures were not. The dominance of *Extrinsic Personal Orientation* probably reflects the fact that scores on the scale represent the extent to which religious belief serves as a buffer against the effects of life’s difficulties. The items focus on protection, comfort, and peace. Although social interactions tend to be pleasant, social interactions do not occur in a vacuum, and the extent to which an individual can cope with stressors that may not be related to interaction per se may influence how they react to the social interactions they have.

Same-sex social activity was positively related to scores on the *CO* scale. As discussed previously, the social environment in which the study was conducted was predominantly Christian. Participants with higher *CO* scores were more likely to find similar others than participants with lower *CO* scores, something that could be particularly important for the types of everyday, causal social contacts that same-sex contacts are more likely to represent compared to opposite-sex contacts.

In contrast, opposite-sex social activity was negatively related to *External Social Orientation* scores (e.g., attending religious services to meet people). This relationship suggests that participants who had a more active opposite-sex social life did not think of religious services as a social venue as much as participants who had a less active opposite-sex social life. Perhaps, individuals with a stronger *External Social Orientation* were attending religious services as an attempt to enhance or expand their opposite-sex social lives compared to individuals with a weaker *External Social Orientation*. Unfortunately, the present study cannot answer such questions.

### Null Relationships

Null findings raise the possibility of type II errors. In terms of finding correlations between the trait-level measures, a sample of 250 provides a power of .9 to detect a correlation of G*Power (Faul et al. 2009). This means that it is likely that the nonsignificant correlations that were found between religiosity and depression were the result of the fact these measures were, in fact, uncorrelated (at least at .2 or more). In terms of relationships between religiosity and reactions to social interactions, using the techniques described by Nezlek and Mroziński (2020), the present design provided a power of approximately .80 to detect relationships between mean reactions to interactions and *Extrinsic Social Orientation* that were comparable in size to the relationships found between mean reactions and *Extrinsic Personal Orientation*. This means that it is likely that the nonsignificant relationships that were found between religiosity and social interaction were the result of the fact these measures were unrelated.

### Integration with Previous Research and Implications

To my knowledge, the present study is the first to examine relationships between religiosity and people’s experience in their day-to-day social interactions, which makes it difficult to integrate the present findings closely with previous research. For example, Lim and Putnam (2010) found a positive relationship between the size of people’s congregational networks and their satisfaction with life. See Krause (2008) for similar findings and a broader discussion of relationships between religious social life and health. Although such findings concern relationships among religiosity, social life, and well-being, the levels of analysis and the constructs that were measured were very different than those in the present study. Most importantly, previous research does not address the roles that religiosity may play in everyday social interaction, interactions that do not necessarily involve people who are members of the same religious organization or perhaps even followers of the same faith.

*Extrinsic Personal Orientation* was the aspect of religiosity that was related most reliably to people’s experiences in social interaction. Although the title of this subscale is technically accurate, the scale measures a personal benefit people derive from religious belief, and the title does not represent the fact that the scale measures the extent to which religious faith provides a source of support for people. The three items on the scale are: “What religion offers me most is comfort in times of trouble and sorrow,” “I pray mainly to gain relief and protection,” and “Prayer is for peace and happiness,” with a response scale of disagree–agree.

These items clearly refer to how much religious beliefs and activities provide a “safe haven” for people. Assuming this is the case, individuals who endorse these items more strongly may enter social interactions with more self-assurance compared to those who endorse them less strongly, a possibility directly supported by the positive relationship between *Extrinsic Personal Orientation* and confidence in interaction. In turn, such self-assurance may translate into more enjoyment. Along the same lines, if people can find comfort in their religious beliefs and activities, this may reduce the negative consequences of negative aspects of interactions (e.g., arguments).

Regardless of the specific mechanisms, to my knowledge, the present results are the first to demonstrate a direct relationship between religiosity and non-religious social experience. Moreover, these results suggest that religious beliefs provide a type of support for people in their day-to-day lives. Interestingly, this support does not seem to be provided by what some would describe as belief per se. Scores on the *Intrinsic Orientation* scale were not reliably related to people’s reactions to their interactions. Rather, it seems that the support people may feel reflects their beliefs about the extent to which activities such as prayer can provide comfort.

## Limitations and Future Directions

Although the results of the present study were clear, they must be considered preliminary. I found an empirical relationship, but the exact mechanisms underlying this relationship could only be inferred. Further research will be needed to explicate such mechanisms.

Clearly, the generalizability of the results of the present study is limited by the nature of sample and the specific methods and measures that were used. Participants in the study were American collegians, and it is an open question as to whether or not the relationships found in the present study would be found in a different sample, for example older adults residing in the community or members of a non-Western society. Moreover, relationships between well-being and religiosity measured using other methods might produce different results.

The present study was essentially correlational in nature, which raises questions about the direction of causality. Does religiosity influence the quality one’s social interactions or vice versa? For example, Nezlek (2001a) found that social interaction was more of an influence on social skills than the reverse. Although it might be possible to assume that the quality of one’s social interactions influences one’s feelings of religiosity, religiosity seems to be an individual difference that reflects a broader and deeper set of influences such as socialization than social skills. Regardless, the present studies suggest that religiosity may influence people’s daily social interactions, and I believe this provides a starting point for further investigation.
